# A 5‐week randomized clinical evaluation of a novel electric toothbrush head with regular and tapered bristles versus a manual toothbrush for reduction of gingivitis and plaque

**DOI:** 10.1111/idh.12372

**Published:** 2018-12-19

**Authors:** Renzo Alberto Ccahuana‐Vasquez, Ralf Adam, Erinn Conde, Julie M. Grender, Pamela Cunningham, C. Ram Goyal, Jimmy Qaqish

**Affiliations:** ^1^ Clinical Operations Procter & Gamble Kronberg Germany; ^2^ Clinical Operations Procter & Gamble Mason Ohio; ^3^ Clinical Statistics and Data Management Procter & Gamble Mason Ohio; ^4^ All Sum Research Center Ltd Mississauga Ontario Canada

**Keywords:** dental plaque, gingivitis, toothbrushing

## Abstract

**Objective:**

To evaluate the efficacy of an oscillating‐rotating (O‐R) electric rechargeable toothbrush with a novel round brush head comprised of regular and tapered bristles in reducing plaque and gingivitis versus a manual toothbrush.

**Methods:**

This was a randomized, examiner‐blind, parallel group, five‐week study. Participants with mild‐to‐moderate plaque and gingivitis received an oral examination and were evaluated for baseline plaque (Rustogi Modified Navy Index), gingivitis (Modified Gingival Index) and gingival bleeding (Gingival Bleeding Index). Qualifying participants were randomly assigned to the novel Oral‐B sensitive brush head (EB60) on an Oral‐B Vitality O‐R handle (D12) or an ADA manual toothbrush. Participants brushed twice daily with the assigned brush and a standard fluoride dentifrice for 5 weeks before returning for an oral examination and plaque and gingivitis evaluations.

**Results:**

A total of 150 participants were randomized to treatment and completed the study (mean age = 45.7 years). Both brushes demonstrated a statistically significant reduction in number of bleeding sites versus baseline (*P* < 0.001). At Week 5, the number of bleeding sites was reduced from baseline by 11.15 (52.2%) for the O‐R brush and 5.04 (23.6%) for the manual brush. The treatment difference was statistically significant (*P* < 0.001). Significant reductions versus baseline (*P* < 0.001) were also seen for both brushes for MGI, GBI and Rustogi plaque measures (whole mouth, gingival margin and proximal), but the O‐R brush produced significantly greater reductions versus the manual brush (*P* < 0.001).

**Conclusion:**

The O‐R handle and round brush head with tapered and regular bristles produced greater plaque and gingivitis reductions than the manual brush.

## 1 INTRODUCTION

Periodontal disease is multifactorial, but typically involves an inflammatory response to dental plaque.^1^, ^2^ It is prevalent, with approximately 80% of the population experiencing gingivitis, the earliest stage of periodontal disease.^3^ Gingivitis develops within days of dental plaque accumulation, and if not reversed may progress to periodontitis with clinical attachment loss.^4^ Preventing gingivitis is a key factor in oral health, therefore effective oral hygiene with thorough plaque removal and control is essential.^5^ Among mechanical methods for plaque removal, the toothbrush is the most frequently used device.

A multitude of manual and electric (ie, power) brush designs are now available, including brushes with advanced technologies designed to improve mechanical plaque removal and the brushing experience. The oscillating‐rotating (O‐R) brush technology has been thoroughly researched and systematic reviews show it is more effective for removing dental plaque and reducing gingivitis than a manual brush.^6^ There is also evidence that O‐R brushes reduce plaque and gingivitis more than sonic brushes in the short term.^7^

Many advanced toothbrush models include innovations in bristle design, such as end rounded, angled, multi‐level and CrissCross^®^ bristle arrangements along the brush head. Tapered bristles (also known as super thin or ultrathin bristles) have also been incorporated into manual brush designs to better reach proximal and other hard‐to‐reach areas,^8^ while providing a gentle brushing experience.^9^, ^10^ Tapered bristles are more flexible and much thinner than others, and have an extended taper. The plaque removal efficacy of tapered bristles in a manual brush design has been shown in in vitro and in vivo studies.^9^, ^11^, ^12^, ^13^, ^14^, ^15^, ^16^, ^17^, ^18^, ^19^

Recently, a round brush head for O‐R electric rechargeable brushes has been developed and designed according to consumer preferences for a gentle brushing experience with a mix of regular end‐rounded and tapered bristles. The purpose of the current study was to evaluate the efficacy of an O‐R brush handle with the novel round brush head comprised of tapered and regular bristles in the reduction of dental plaque and gingivitis versus a manual toothbrush among generally healthy participants with mild‐to‐moderate plaque and gingivitis over a 5‐week period. This is the first reported research on the new brush head.

## 2 METHODS

### 2.1 Study design

This was a five‐week, single‐centre, randomized, two‐treatment, examiner‐blind, parallel group study. Prior to starting the study, Institutional Review Board approval was obtained for the study protocol and informed consent form (16048‐11:57:4531‐05‐2016). One hundred and fifty‐two potential participants were recruited by All Sum Research Center Ltd. in Mississauga, Ontario by phone or email in April of 2016. Participants signed a written informed consent prior to their participation in the study. Qualified participants were instructed to abstain from brushing and performing any oral hygiene 3‐6 hours prior to their Baseline and Week 5 visits, and to abstain from eating, chewing gum or drinking after oral hygiene on the morning of their appointments (small sips of water were allowed up until 45 minutes prior to their appointment). At the baseline visit, participants first received an oral examination. This was followed by an assessment of gingivitis using the Modified Gingival Index (MGI) and Gingival Bleeding Index (GBI).^20^, ^21^ Next, an assessment of plaque was performed using the Rustogi Modified Navy Plaque Index (RMNPI) after plaque on all surfaces was first stained using Chrom‐O‐Red erythrosine disclosing solution (Germiphene Corp., Bradford, ON, Canada).^22^

#### 2.1.1 Study population

Participants had to be typical manual toothbrush users 18 years of age or older, in good general health, without orthodontic appliances, and have a minimum of 16 natural teeth with facial and lingual scorable surfaces for consideration. Teeth with scorable surfaces excluded third molars, teeth (or implants) with crowns or bridges, and teeth with large restorations covering >50% of the tooth surface. Participants qualified for entrance into the study if they met these requirements and had a baseline gingivitis (MGI) score of at least 1.75 but not greater than 2.3, a minimum of 10 bleeding sites (GBI score of 1 or 2), and a whole mouth RMNPI score greater than 0.50.

#### 2.1.2 Randomization and treatments

Qualified participants (n = 150) were stratified on baseline scores for MGI (≤2.0 vs >2.0), number of bleeding sites (≤22.0 vs >22.0), whole mouth mean RMNPI (≤0.65 vs >0.65) and tobacco use (yes/no). Participants were then randomly assigned to one of two treatment groups based on a computer‐generated schedule provided by the study sponsor, such that an approximately equal number of participants were assigned to each treatment group within each of the specified strata.

**Figure 1:**
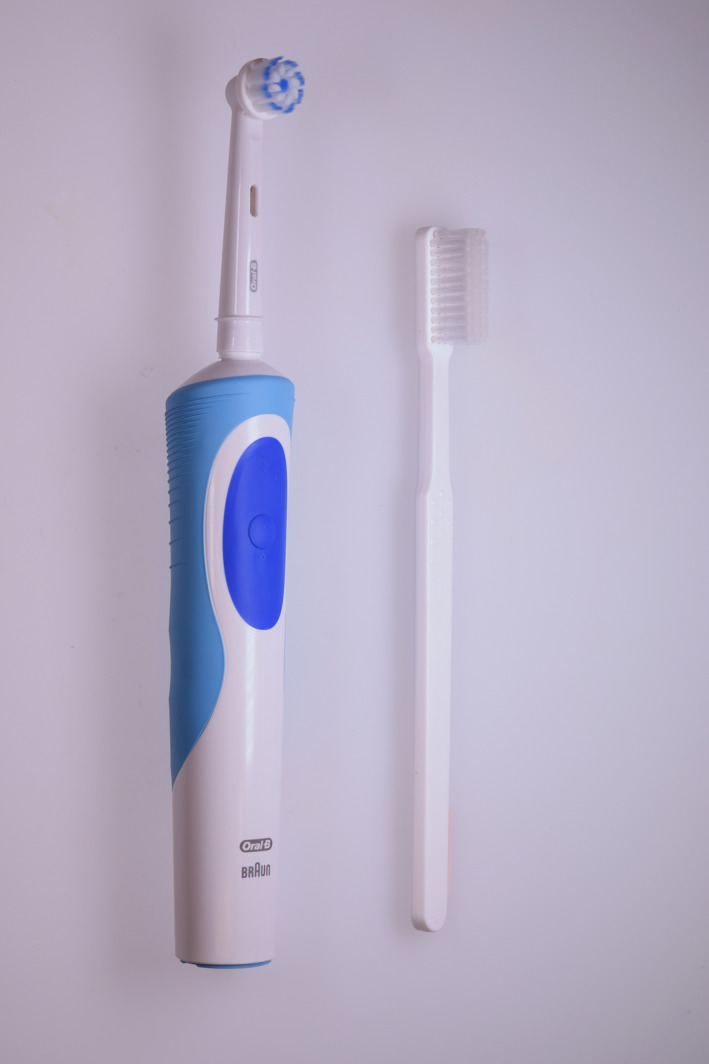
Test products. Left: Oral‐B Vitality brush handle and round brush head with regular end‐rounded bristles (centre ring) and tapered bristles (outer ring). Right: Manual toothbrush

- An O‐R electric rechargeable toothbrush handle (Oral‐B Vitality, D12) and round brush head (marketed as Oral‐B Sensi Ultrathin or Oral‐B Pro Gum Care depending on the region, EB60; Procter & Gamble, Cincinnati, OH, USA) in which the centre tufts of bristles are standard, end‐rounded bristles, while the outer ring of tufts consists of tapered bristles that are standard diameter at the base and taper to a fine tip (0.01 mm diameter at the tip); or
- An ADA manual toothbrush (American Dental Association, Chicago, IL, USA) with end‐rounded bristles (Figure 1).

Both groups brushed with the same standard sodium fluoride dentifrice (Crest® Cavity Protection, Procter & Gamble, Cincinnati, OH, USA).

Participants received their assigned products by clinical site personnel in a separate area to ensure the examiner was blinded to treatment assignment. In the same area, they then received verbal and written instructions on oral hygiene and product usage, and were asked to perform a supervised brushing in front of a mirror using the assigned products. Participants using the electric brush were instructed to brush for 2 minutes, twice daily for approximately five weeks with their assigned toothbrush and dentifrice according to manufacturer's usage instructions. Participants using the ADA manual brush were instructed to brush in their usual manner with the assigned products. At the Week 5 visit, all participants were ascertained to still meet the study criteria, including having refrained from brushing for 3–6 hours prior to their appointment, and from eating, chewing gum and drinking as described above. Each participant then received an oral examination, MGI, GBI and RMNPI plaque assessments as described for the Baseline visit.

#### 2.1.3 Clinical assessments

All clinical assessments were performed by the same experienced examiner^23^, ^24^ at the baseline and Week 5 visits. The safety assessment included visual examination of the intra‐oral and oropharyngeal soft tissues, lips and the peri‐oral area using a standard dental light, dental mirror and gauze. The dentition was examined using a standard dental light, dental mirror and air syringe.

The MGI gingivitis evaluations^21^ were conducted by scoring gingival inflammation on up to six sites (distobuccal, buccal, mesiobuccal, mesiolingual, lingual and distolingual) on all scorable teeth. A scale of 0‐4 was used as follows: 0 = normal (absence of inflammation); 1 = mild inflammation (slight change of colour, little change in texture) of any portion of, but not the entire, marginal or papillary gingival area; 2 = mild inflammation of the entire gingival area; 3 = moderate inflammation (moderate glazing, redness, oedema, and/or hypertrophy) of the marginal or papillary gingiva area; and, 4 = severe inflammation (marked redness and oedema/hypertrophy, spontaneous bleeding, or ulceration) of the marginal or papillary gingival area.

Whole mouth MGI scores for each participant were obtained by summing all scores and dividing the total by the number of scored sites at each examination.

The GBI evaluations were conducted in the manner defined by Saxton and van der Ouderaa.^20^ After lightly air drying the area, a periodontal probe with a 0.5 mm diameter tip was inserted into the gingival crevice to a depth of 2 mm or until slight resistance was felt. While maintaining contact with the sulcular epithelium, the probe was then run gently around the tooth at an angle of approximately 60°. To avoid undue penetration into the tissue, minimum axial force was used and the probe was moved around the crevice gently stretching the epithelium. Each gingival area (buccal mesial, buccal distal and lingual) was probed in this manner, waiting approximately 30 seconds before recording the number of areas that bled in accordance with the following scale: 0 = absence of bleeding after 30 seconds; 1 = bleeding observed after 30 seconds; and, 2 = immediate bleeding observed. GBI whole mouth scores for each participant were computed by summing the scores and dividing the total by the number of scored sites at each examination. The number of bleeding sites was determined by counting the number of probed sites that bled (sites with a GBI score of 1 or 2).

The final step involved plaque evaluation using the Rustogi Modification of the Navy Plaque Index (RMNPI).^22^ Plaque was scored as absent (0) or present (1) on up to nine tooth areas (A‐I) for both buccal and lingual surfaces. The Mean Plaque Index (MPI) was computed by summing the total number of tooth areas with plaque present and dividing this by the total number of tooth areas scored. Areas (A‐I) were calculated for the whole mouth MPI, areas A‐C for the gingival margin MPI and areas D and F for the proximal region MPI.

#### 2.1.4 Data analysis and statistical methods

The sample size was determined by power analyses with *α* = 0.05, using a 2‐sided test. Based on whole mouth MGI variability of 0.084 and whole mouth RMNPI variability of 0.042, it was determined that a sample size of 75 participants in each group would provide 90% power to detect a difference in mean MGI and RMNPI scores of 0.050 and 0.025 units, respectively, between treatments. Statistical analyses were performed to determine group differences. A two‐sample t test was used to compare group differences for age; gender was compared using a chi‐square test, and for ethnicity and smoking status using Fisher's exact test. Separate calculations were performed at Baseline and Week 5 to determine whole mouth, gingival margin and proximal region RMNPI scores, as well as MGI, GBI and number of bleeding site scores. Analyses for gingivitis efficacy were based on the mean changes in MGI, GBI and number of bleeding sites from baseline to Week 5, and plaque efficacy analyses were based on the mean changes in RMNPI over the same time period. An analysis of covariance (ANCOVA) was performed to determine treatment differences on the plaque reduction with the respective baseline whole mouth and proximal RMNPI scores as the covariates. Similar analyses were carried out for each gingivitis endpoint (MGI, GBI and number of bleeding sites), with MGI being primary. For gingival margin RMNPI, an ANOVA was conducted since the baseline plaque scores were 1.0 for all participants in the gingival margin area. All statistical tests for treatment comparisons were two‐sided with a significance level of *α* = 0.05.

## 3 RESULTS

**Figure 2:**
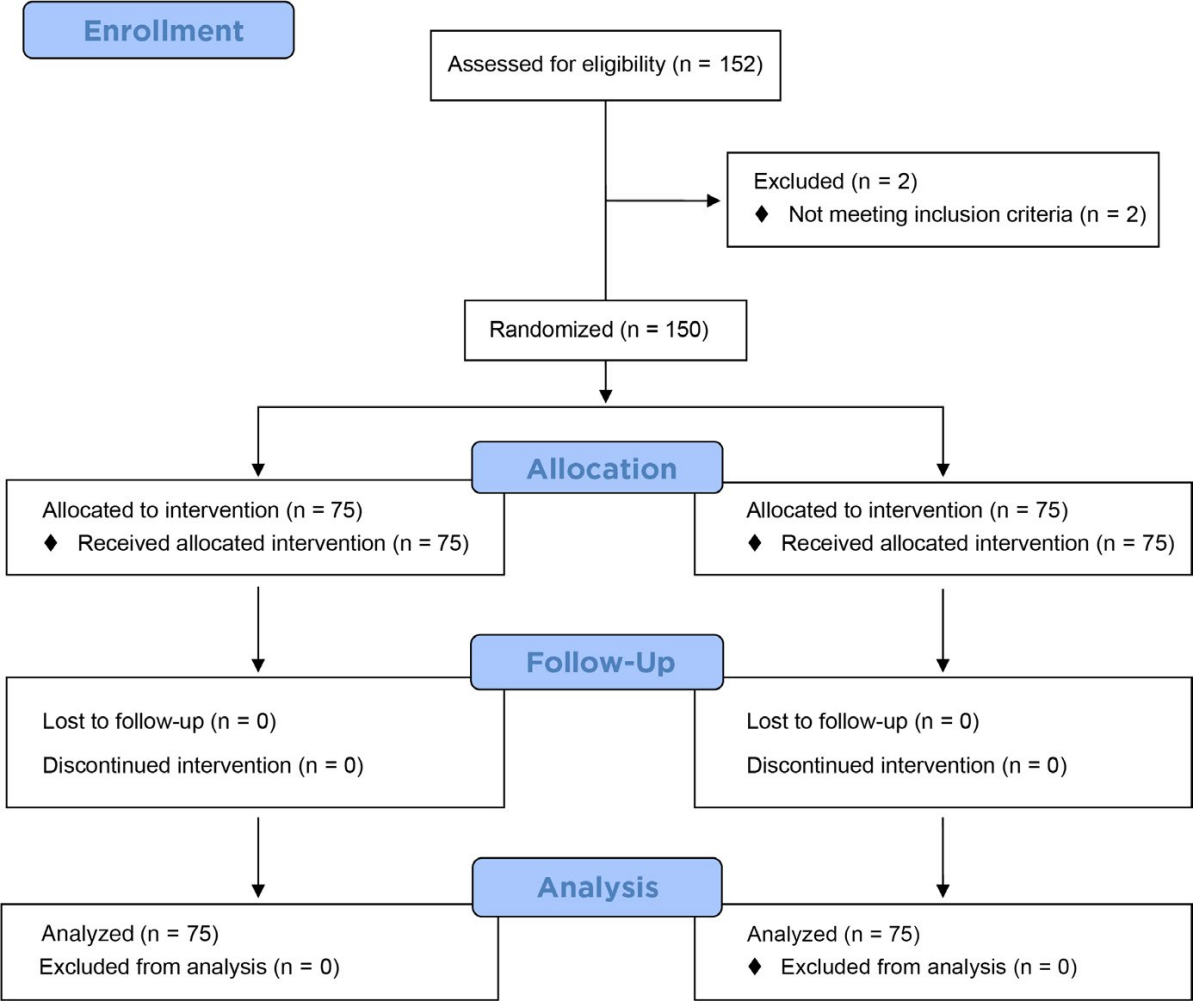
Flow diagram

A total of 150 participants were randomized to treatment; all participants completed the study (Figure 2). The mean age of participants was 45.7, with ages ranging from 18 to 77 years. Overall, 64% of participants were female, and 92% were nonsmokers. Statistical analyses showed that the treatment groups were well‐balanced for age, gender and smoking status (*P* ≥ 0.496, Table 1).

**Table 1:** Demographic characteristics of study participants (randomized participants)

Demographic characteristics	ADA manual (n = 75)	O‐R brush with novel head (n = 75)	Total (n = 150)
Age (Y)^a^			
Mean	45.5	45.9	45.7
SD	12.93	12.94	12.89
Minimum	19	18	18
Maximum	77	70	77
Gender^b^,^c^			
Female	50 (66.7%)	46 (61.3%)	96 (64.0%)
Male	25 (33.3%)	29 (38.7%)	54 (36.0%)
Smoker^b^,^c^			
Yes	6 (8.0%)	6 (8.0%)	12 (8.0%)
No	69 (92.0%)	69 (92.0%)	138 (92.0%)

a Two‐sample t test was used to compare mean age between the two treatment groups (*P* = 0.855).

b Number and per cent of participants in each category.

c Chi‐square test was used to assess gender balance between the two groups (*P* = 0.496).

### 3.1 MGI, GBI and number of bleeding sites

The baseline means and Week 5 reductions in MGI, GBI and number of bleeding sites are shown in Table 2. There were no statistically significant differences at baseline between groups for whole mouth MGI or GBI scores (*P* ≥ 0.575*)*. At Week 5, both brushes showed a statistically significant reduction in MGI and GBI scores compared to baseline (*P* < 0.001). MGI was reduced by 13.1% for the O‐R group and 5.4% for the manual group and GBI was reduced by 54.0% and 23.3% for the O‐R and manual groups, respectively. Statistically significantly greater reductions were observed for the O‐R brush versus the manual brush for MGI and GBI measures (*P* < 0.001).

**Table 2:** Baseline mean and Week 5 MGI, GBI and number of bleeding sites reduction^a^

	Baseline Mean^b^ (SD)	Week 5 Adj. mean reduction (SE)^c^	% change from baseline	Between‐brush difference	Between‐brush difference *P*‐value
Modified Gingival Index					
O‐R brush/novel head	2.096 (0.0813)	0.275 (0.0106)	13.1%	0.161	*P* < 0.001
ADA manual	2.104 (0.0880)	0.114 (0.0106)	5.4%		
Gingival Bleeding Index					
O‐R brush/novel head	0.146 (0.0957)	0.081 (0.0030)	54.0%	0.046	*P* < 0.001
ADA manual	0.152 (0.1173)	0.035 (0.0030)	23.3%		
Number of Bleeding Sites					
O‐R brush/novel head	20.72 (12.627)	11.15 (0.404)	52.2%	6.112	*P* < 0.001
ADA manual	22.00 (14.505)	5.04 (0.404)	23.6%		

a N = 75 per group.

b P‐value for treatment group comparison at baseline was 0.575 for MGI, 0.729 for GBI and 0.565 for number of bleeding sites.

c All changes from baseline were statistically significant, *P* < 0.001.

The mean number of bleeding sites for the O‐R and manual brush groups at baseline were 20.72 and 22.00, respectively, with no statistically significant difference between groups (*P* = 0.565). Both groups showed a statistically significant reduction in number of bleeding sites relative to baseline (*P* < 0.001). Mean bleeding site reductions from baseline at Week 5 were 11.15 (52.2%) for the O‐R brush and 5.04 (23.6%) for the manual brush; the reduction was statistically significantly greater for the O‐R brush compared to the manual brush group (by 6.11, *P* < 0.001).

### 3.2 Plaque scores

Randomized participants presented at baseline with moderate plaque accumulations (whole mouth RMNPI >0.50). Baseline whole mouth RMNPI scores were 0.633 for the O‐R group and 0.625 for the manual group, with no statistically significant difference between the groups for any baseline plaque measure (whole mouth, proximal, gingival margin; *P* ≥ 0.246). At Week 5, statistically significant reductions in whole mouth, gingival margin and proximal region RMNPI scores were observed for both groups (*P* < 0.001 for each measure) (Table 3). For all three RMNPI measures, the results statistically significantly favoured the O‐R brush over the manual brush (*P* < 0.001 for each).

**Table 3:** Baseline mean plaque and Week 5 plaque reductions from baseline^a^

	Baseline Mean^b^ (SD)	Week 5 Adj. mean reduction (SE)^c^	% change from baseline	Between‐brush differences	Between‐brush differences *P*‐value
Whole mouth plaque					
O‐R brush/novel head	0.633 (0.0486)	0.127 (0.0048)	20.2%	0.064	*P* < 0.001
ADA manual	0.625 (0.0409)	0.064 (0.0048)	10.1%		
Gingival margin plaque					
O‐R brush/novel head	1 (0)	0.043 (0.0035)	4.3%	0.029	*P* < 0.001
ADA manual	1 (0)	0.014 (0.0035)	1.4%		
Proximal region					
O‐R brush/novel head	0.998 (0.0152)	0.235 (0.0162)	23.6%	0.152	*P* < 0.001
ADA manual	0.994 (0.0293)	0.083 (0.0162)	8.3%		

a N = 75 per group.

b *P*‐value for treatment group comparison at baseline was 0.246 for whole mouth and 0.331 for proximal region plaque.

c All changes from baseline were statistically significant, *P* < 0.001.

### 3.3 Safety

No adverse events were observed or reported during the study.

## 4 DISCUSSION

Manual and electric brush designs have improved as a result of research on brush handles, brush heads and bristle configurations. Tapered bristles represent one such improvement whereby thinner, more flexible bristles can remove dental plaque, including in hard‐to‐reach areas,^8^ while providing a gentle brushing experience.^9^, ^10^ Tapered bristles may also deliver functional chemistry in dentifrice to hard‐to‐reach areas better than conventional bristles.

The current clinical study is the first reported on a brush head with regular end rounded and tapered bristles on an O‐R handle. In comparison to the manual brush, plaque and gingivitis improvements were significantly greater for the electric O‐R brush. The increased plaque removal efficacy of the O‐R brush was especially marked for proximal areas, where long, thin tapered bristles have been shown to have cleaning advantages.^8^

Since an electric toothbrush head control with only end‐rounded bristles was not included in this trial, it is not possible to ascertain the relative efficacy contribution of the electric brush technology relative to the tapered bristles. It is possible to make broad comparisons based on a large systematic review evaluating the plaque‐ and gingivitis‐reducing effects of electric toothbrushes collectively versus manual toothbrushes.^6^ The review included fifty‐five clinical trials, twenty‐seven of which evaluated oscillating‐rotating electric toothbrushes using various handles, brush heads and clinical indices. Looking across the 1‐ to 3‐month studies, electric toothbrushes were found to have an 11% advantage versus manual for plaque removal a 6% benefit for gingivitis reduction. In this 5‐week trial, the O‐R toothbrush with the novel head comprised of tapered and end‐rounded bristles showed a 10% plaque removal advantage and an 8.5% gingivitis reduction advantage versus the manual toothbrush.

Previous in vitro research compared different designs of manual toothbrushes with tapered bristles regarding subgingival access efficacy and results showed that manual brushes with highly tapered bristles were statistically significantly (*P* < 0.01) more effective reaching the subgingival area than the conventional toothbrush with slightly tapered bristles.^15^ Other in vitro tests proved that the combination of regular end‐rounded bristles and tapered bristles in manual toothbrushes was statistically significantly (*P* < 0.01) more effective in removing artificial plaque material on the fissure area of occlusal surfaces than brushes with only regular bristles.^14^

A recent systematic review and meta‐analysis compared the effect of tapered toothbrushes bristles on dental plaque removal and gingivitis reduction.^9^ Seven clinical studies which assessed manual toothbrushes were included in this review. The authors concluded that there is not enough evidence showing that tapered bristles remove more plaque than regular bristles. Regarding gingivitis reduction, the meta‐analysis showed a significant gingivitis reduction in favour of tapered bristles; however, the evidence was considered minimal. The absence of robustness for the gingivitis results could be a consequence of the small number of clinical trials performed for this assessment. In addition, a recent publication summarizing two clinical trials including marketed controls with regular end‐rounded bristles showed that a manual brush (Oral‐B Super Thin Indicator toothbrush, OM159; Procter & Gamble, Cincinnati, OH, USA) with the same tapered bristles included in the electric brush head evaluated in this trial provided significant plaque removal and gingivitis reductions.^16^

The findings of this clinical trial can be extrapolated to the representative population. Participants were usual manual toothbrush users with mild‐to‐moderate plaque and gingivitis, which represents a large percentage of the population. The reduction in the number of bleeding sites for the O‐R group appears to be clinically meaningful given a reduction in bleeding is an important indicator of improvement in gingival health for both professionals and patients.^25^ One limitation of this trial relative to the literature is the lack of participant experience data. In other research, patients have reported finding a manual toothbrush with tapered bristles to be gentle and more pleasant to use.^9^ Post‐surgical patients (eg, wisdom tooth extractions) in particular have reported the brushing experience to be gentle, noting they prefer a manual brush with tapered bristles versus a manual brush with end‐rounded bristles.^9^ A systematic review found levels of gingival abrasion similar to those for end‐rounded brushes,^9^ while individual studies,^10^, ^18^, ^26^ including those with post‐surgical patients,^10^ have shown lower levels of gingival abrasion for tapered bristle brushes than for standard control brushes, in one case 85.5% and 73.9% lower at 6 and 12 weeks, respectively. The safety and gentleness of O‐R brushes in general have been confirmed in systematic reviews and long‐term studies, up to 3 years.^28^, ^29^

This study focused on the therapeutic effects of the new brush head on an entry‐level handle among usual manual toothbrush users. Future research could involve evaluations of the brush head on more advanced handles, comparisons versus different toothbrush controls, assessments among typical electric toothbrush users and/or a survey of patient experience.

## 5 CONCLUSIONS

The current study demonstrates the efficacy of a novel round sensitive brush head with tapered bristles on an O‐R electric toothbrush handle. In comparison to a standard manual brush with end‐rounded filaments, the O‐R brush resulted in significantly greater reductions in gingival inflammation, number of bleeding sites and plaque. This brush head and handle combine the proven efficacy of the O‐R technology with tapered bristles to provide effective cleaning, particularly in hard‐to‐reach proximal areas.

## 6 CLINICAL RELEVANCE

### 6.1 Scientific rationale for study

Tapered bristles, used in manual toothbrushes to reduce plaque and gingivitis, have been incorporated with regular bristles in a round brush head to provide efficient plaque removal. This study evaluated the efficacy of the new head in reducing plaque and gingivitis on an entry‐level O‐R handle versus a manual brush.

### 6.2 Principal findings

The brush head and O‐R handle provided greater plaque and gingivitis reductions than the manual brush.

### 6.3 Practical implications

The new brush head with tapered and end‐rounded bristles is an effective option for patients with poor plaque control or gingivitis.
